# Prioritizing spatial accuracy in high-resolution fMRI data using multivariate feature weight mapping

**DOI:** 10.3389/fnins.2014.00066

**Published:** 2014-04-16

**Authors:** Johannes Stelzer, Tilo Buschmann, Gabriele Lohmann, Daniel S. Margulies, Robert Trampel, Robert Turner

**Affiliations:** ^1^Department of Neurophysics, Max Planck Institute for Human Cognitive and Brain SciencesLeipzig, Germany; ^2^Danish Research Centre for Magnetic Resonance, Copenhagen University Hospital HvidovreHvidovre, Denmark; ^3^Department of Diagnostics, Fraunhofer Institute for Cell Therapy and ImmunologyLeipzig, Germany; ^4^Department of Biomedical Magnetic Resonance, University Hospital TübingenTübingen, Germany; ^5^Magnetic Resonance Center, Max Planck Institute for Biological CyberneticsTübingen, Germany; ^6^Max Planck Research Group for Neuroanatomy and Connectivity, Max Planck Institute for Human Cognitive and Brain SciencesLeipzig, Germany

**Keywords:** fMRI, MVPA, searchlight, nonparametric statistics, decoding

## Abstract

Although ultra-high-field fMRI at field strengths of 7T or above provides substantial gains in BOLD contrast-to-noise ratio, when very high-resolution fMRI is required such gains are inevitably reduced. The improvement in sensitivity provided by multivariate analysis techniques, as compared with univariate methods, then becomes especially welcome. Information mapping approaches are commonly used, such as the searchlight technique, which take into account the spatially distributed patterns of activation in order to predict stimulus conditions. However, the popular searchlight decoding technique, in particular, has been found to be prone to spatial inaccuracies. For instance, the spatial extent of informative areas is generally exaggerated, and their spatial configuration is distorted. We propose the combination of a non-parametric and permutation-based statistical framework with linear classifiers. We term this new combined method Feature Weight Mapping (FWM). The main goal of the proposed method is to map the specific contribution of each voxel to the classification decision while including a correction for the multiple comparisons problem. Next, we compare this new method to the searchlight approach using a simulation and ultra-high-field 7T experimental data. We found that the searchlight method led to spatial inaccuracies that are especially noticeable in high-resolution fMRI data. In contrast, FWM was more spatially precise, revealing both informative anatomical structures as well as the direction by which voxels contribute to the classification. By maximizing the spatial accuracy of ultra-high-field fMRI results, global multivariate methods provide a substantial improvement for characterizing structure-function relationships.

## Introduction

The advent of functional magnetic resonance imaging (fMRI) at ultra-high-field strengths allows an impressively fine-grained insight into human cortical function. Modern scanners at 7T or higher allow researchers to resolve functional data at ever finer spatial scales, even to the point of resolving individual gray matter layers (Polimeni et al., 2010; Trampel et al., 2011). The benefits of improved resolution are accompanied by new challenges, however, particularly with regard to data analysis, as it is not obvious which analysis technique may best take advantage of the richer data. For instance, classical activation-based approaches such as the general linear model (Poline and Brett, 2012) generally rely on spatial smoothing for statistical correction for multiple comparisons, and hence are unable to make appropriate use of the high resolutions. While a more sophisticated approach has been proposed (Harrison et al., 2008) this is computationally laborious and does not have face validity in terms of actual neuroanatomy. A more promising means of exploiting higher resolution is multivariate pattern recognition analysis (MVPA), which enables fine-grained components of the brain activity signal to contribute relevantly (Norman et al., 2006). It is often desirable to map the spatial location of discriminating patterns, or in other words, where in the brain information about the experimental condition is present.

For this, “information mapping” methods, such as the “searchlight” approach are often employed (Kriegeskorte et al., 2006). The searchlight method attempts to extract the predictive power of a small neighborhood of voxels (the *searchlight*) with regards to the stimulus condition, and maps the result of the analysis back to the center voxel of the searchlight. Repeating this procedure over all locations yields an information map charting the presence and location of information relating to stimulus condition. It should be noted that the searchlight method is essentially a *local* multivariate pattern recognition technique that fails to take into account globally distributed voxel patterns.

Alternatively, *global* information maps can be computed without such spatial preselection of voxels using multivariate classifiers with support for high dimensional data or by using dimensionality-reduced brain data (e.g., by first performing a principal component analysis). Appropriate classifiers provide information on the contribution of individual features (i.e., voxels or principal components) to the classification decision. Mapping this influence back onto the voxel space allows generation of a whole-brain information map (Mourão-Miranda et al., 2005), which delineates the discriminative volume.

Previously, the searchlight method has been reviewed critically on the grounds of interpretability and with regard to spatial inaccuracies in the searchlight information maps which obscure the true local information content (Viswanathan et al., 2012; Etzel et al., 2013). These shortcomings form the greatest concern with very high spatial resolution fMRI data, such as that obtainable at ultra-high-field, as they may well negate the gain in higher spatial resolution. In particular, the lower voxel-wise signal-to-noise ratio at very high resolutions requires larger searchlight diameters to obtain significant results, exacerbating spatial inaccuracies.

In the present work, we investigate the quality of the searchlight method as a tool for the analysis of ultra-high-field fMRI data. As an alternative to the searchlight approach, we present a global multivariate method adapted from previous work (Mourão-Miranda et al., 2005), which we combine with a non-parametric solution for the multiple comparison problem (Stelzer et al., 2013). To our knowledge, this is the first implementation fully accounting for the multiple comparisons problem, tailored for this widely used multivariate framework for brain mapping. We compare these two information-mapping methods as a means for analyzing ultra-high resolution fMRI and simulated data. Noteworthy, while both methods (searchlight and global information maps) incorporate different assumptions and implementations, in research practice the results ultimately are interpreted in a *very similar fashion*: Both approaches provide voxel maps, which delineate voxels containing stimulus-relevant information.

## Materials and methods

### 7T tapping data set

The ultra-high field fMRI study comprised ten healthy subjects (age range 23–28, right-handed). The study was carried out in accordance with the ethics approval from the University of Leipzig and written informed consent was obtained before each study. One single subject was selected as representative for visualization.

Per experimental condition, we conducted 15 trials (26.4 s each) from four experimental conditions. Trials were presented subsequently (not randomized) in a block design. The basic task was self-paced sequential tapping of four fingers of the right hand to the thumb at a frequency of about 2 Hz. The first experimental condition was rest (no movement, no imagination, i.e., base-line condition), followed by the imagined finger movement condition. The third and fourth conditions were finger tapping (tapping of four fingers of the right hand to the thumb) and movement of four fingers as in the previous condition but without thumb contact. Due to limitations in scan time there was no rest period in between subsequent trials. Performance compliance with this easy task was confirmed using video monitoring. For the analysis in our present work, only two conditions were used: Resting (no task, no hand movement, no motor imagination) and sequential tapping with four fingers of the right hand to the thumb of the right hand. (The conditions omitted were: Imagined finger tapping without actual finger movement and finger movement without touching the thumb).

The experiment was performed with a MAGNETOM 7T scanner (Siemens Healthcare, Erlangen, Germany), using a 24-channel head coil (NOVA Medical Inc., Wilmington MA, USA). The functional scans contained 17–31 axial slices (depending on the subject) covering the left motor cortex (*TR* = 3300 ms, *TE* = 25 ms, slice thickness 0.75 mm, in plane resolution 0.75 × 0.75 mm^2^) using a novel acceleration technique (Heidemann et al., 2012).

Head motion correction was carried out using SPM8 (Wellcome Department of Imaging Neuroscience, Institute of Neurology, London, UK). Low frequency drifts were removed using a temporal high-pass filter (*f*_highpass_ = 1/80 *s*^−1^) with LIPSIA (Lohmann et al., 2001). Using LIPSIA, a GLM was fitted to each trial to estimate its β-parameters. We used a gamma-function as hemodynamic response function (Glover, 1999). The GLM yielded 15 three-dimensional β-maps per experimental condition. The β-parameters were estimated on the z-scored fMRI time series data (default settings in LIPSIA) and represent the degree of fit between the trial data and the model.

### Data generation for simulation

A simulated data set of 30 scans (15 each for class A and B) of one “virtual” subject was generated. Each volume (size 66 × 22 × 22 voxels) was filled with Gaussian noise [~ *N*(0, 1)] and smoothed slightly with a Gaussian smoothing kernel (FWHM = 1 voxel). An offset size of 0.5 was added at six locations (three in class A and B, respectively), shaping six half-cubes positioned above and below the centerline of the volume (Figure 3A). Upper half-cubes were class A, lower half-cubes were class B.

The size of the half-cubes varied. The leftmost half-cube had a dimension of 6 × 6 × 1 voxels (fine information spread), the second one had a dimension of 6 × 6 × 2 voxels (medium information spread) and the rightmost half-cube 6 × 6 × 3 voxels (coarse information spread). There was a 4-voxel gap between the leftmost, a 2-voxel gap between the middle, and no gap between the two rightmost half-cubes (see Figure 3A).

### Searchlight decoding

Each voxel of the brain was first scaled to the range [−1, +1]. Around every voxel (*center voxel*), we constructed a spherical *searchlight* that contained every neighbor voxel within a given radius *r* (Kriegeskorte et al., 2006; Stelzer et al., 2013). For every searchlight, we trained and cross-validated (“*leave 2 out*” method) a linear support vector machine (Chang and Lin, 2011). The center voxel was then associated with the mean cross-validation accuracy (i.e., the percentage of correctly predicted labels) and later used for brain visualization.

### Feature weight mapping

In FWM, the whole brain data was first reduced in dimensionality with PCA [dim = *df* = #samples-1 = 29 (Abdi and Williams, 2010)]. The PCA procedure obtained a new representation X^*^ of the data matrix X by orthogonally transforming the columns (features) of X into linearly uncorrelated components (*principal components*). The principal components were sorted in decreasing order by the variance they explain in the data (Abdi and Williams, 2010). The maximal number of principal components was the maximum of the number of observations and features (more precisely the rank of X, dim = 30). In our data sets, the last component contained no substantial variance and was left out. Hence we always employed every PCA component with the exception of the last one, corresponding to the number of maximal degrees of freedom from the matrix decomposition. The PCA projection was calculated by performing a singular value decomposition of X.

The resulting features were scaled to be within the range [−1, +1]. A linear support vector machine was then trained using the entire data set (Chang and Lin, 2011), without the application of further cross-validation procedures. Linear support vector machines divide data samples into their classes by constructing a maximally separating hyperplane between the high-dimensional data points. The hyperplane is established by a set of points $\vec{x}$ and the normal vector $\vec{w}$ to the hyperplane in the formula $\vec{w}$ · $\vec{x}$ −b = 0. The optimal vector w is calculated by minimizing $\left\|\vec{w}\right\|$ in the formula *y_i_* · (*w_i_* · *x_i_* − b)≥ 1 with *i* ϵ {1, ..,*n*} and *x_i_* being the sample vectors from X^*^. The values *w_i_* are the weights given to each feature dimension, (i.e., the principal components), and signify the importance of the component in making the classification decision. We transform the weights of principal components back to weights of individual voxels by reversing the PCA transformation. Note that this procedure solely resulted in weights and not in decoding accuracies.

### Non-parametric statistics

We employed permutation tests for assessing statistical significance (Golland et al., 2005; Mourão-Miranda et al., 2005; Stelzer et al., 2013). No spatial smoothing was applied, however due to interpolations (motion correction etc.) and the biophysical properties of the BOLD signal, a certain level of intrinsic smoothness was present in the data. Permutation tests were carried out by randomly shuffling the order of samples within a data set. For SLD, permutations were assigned before splitting the data into training or test sets to ensure no bias due to uneven class distribution. Each permutation was held fixed for all locations of the searchlight, preserving spatial correlations. For FWM, permutations were assigned on the principal component level.

For each permutation, an accuracy map (SLD) or weights map (FWM) was computed (cf. two previous sections). The empirical *p*-value of each voxel was then the probability of the original accuracy/weight of this voxel in the empirical distribution function given all permutations.

Using the per-voxel *p*-value as a threshold map, we binarized the original and permuted accuracy or weight-maps. For SLD, we employed a one-sided (*lower than p-value*) statistic, for FWM we employed a two-sided [*lower than p/2 or higher than* (*1-p/2*)] statistic.

Counting the cluster sizes (six connectivity scheme) in the permuted binarized maps, we calculated an empirical distribution function (edf) of cluster sizes. Using this edf, we calculated the probability of each cluster in the original binarized maps. In the case of FWM, positive and negative clusters were recovered separately. The final assessment of cluster *p*-values was corrected with FDR (Benjamini, 1999; using a cluster *p*-value of 0.05).

### Analysis of simulation data

The analysis was carried out with and without multiple comparison correction. Without multiple comparisons correction, only *p*-value maps were binarized and no further cluster statistics were computed. The remaining voxels were deemed significant.

With multiple comparison correction, the entire cluster-based analysis (including the empirical cluster-size distribution derived from permuted binarized maps) was repeated for different levels of voxel-wise *p*-value thresholds. Voxels in the remaining clusters were deemed significant.

Precision is defined as the proportion of true positives and all positives:

$$\text{Precision}=\frac{\text{true positives}}{\text{true positives + false positives}}$$

Sensitivity is defined as the proportion of voxels in informative regions that were discovered significant:

$$\text{Sensitivity}=\frac{\text{true positives}}{\text{true positives + false negatives}}$$

## Results

### 7T data set

**Figure 1:**
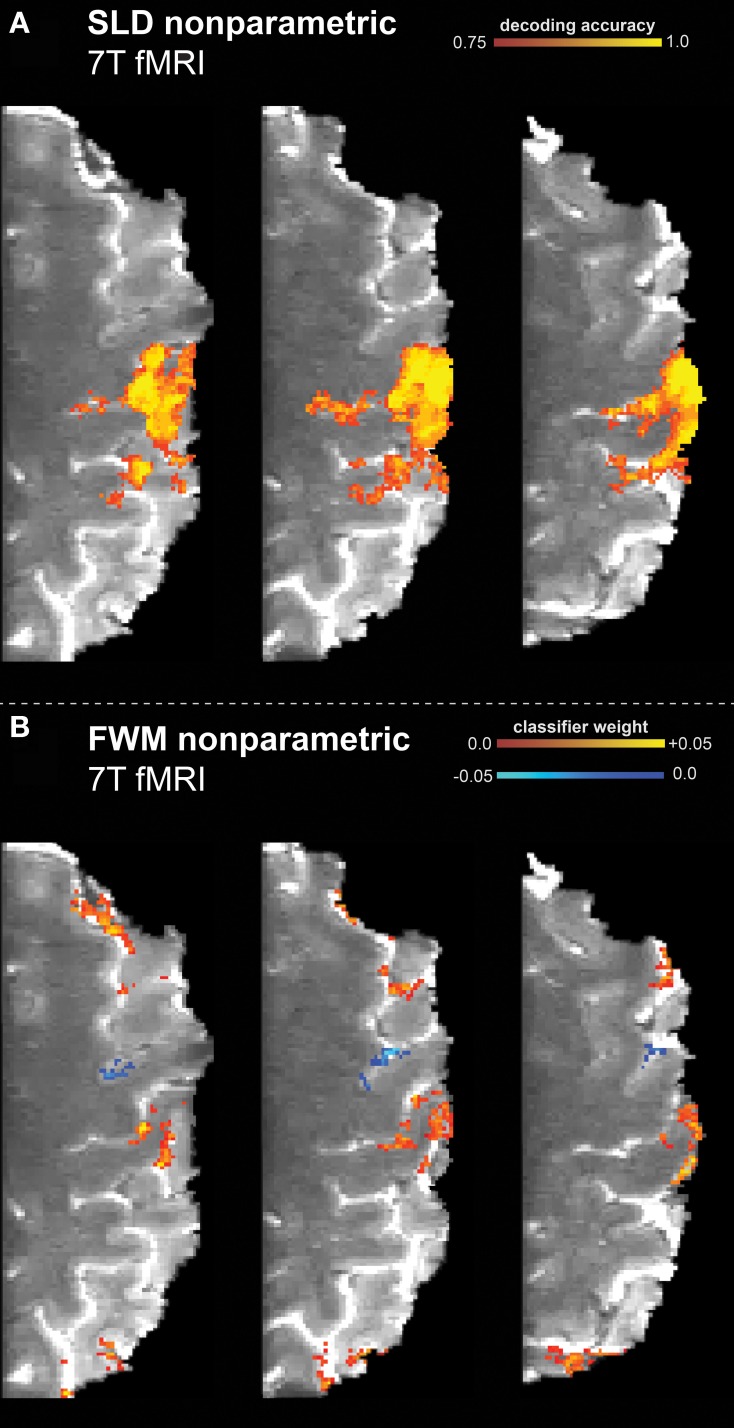
**Results of the high resolution 7T finger tapping data set, classifying resting vs. finger tapping with touch**. The non-parametric framework (including multiple comparison correction) had been applied to the searchlight decoding (SLD) and feature weight mapping (FWM) methods. **(A)** SLD method (diameter = 3.75 mm) with a voxel-wise threshold of *p*_vox_ = 0.001 (one-sided). **(B)** FWM method, using a (two-sided) threshold of *p*_vox_ = 0.05.

The ultra-high resolution 7T finger tapping experiment was analyzed using both the Feature Weight Mapping (FWM) and Searchlight Decoding (SLD) method on a single-subject level. Three axial slices of the results for the analysis are shown in Figures 1A,B respectively using two different thresholds (the appropriate threshold for the respective method was chosen based on simulations in the next section). The searchlight radius was set to 3 mm.

The SLD method found the hand knob part of the motor cortex to be significantly discriminative regarding the stimulus condition (resting vs. finger tapping). Similarly, this region was also labeled as discriminative using the FWM method.

Furthermore, while the SLD method identified the existence and degree of discriminative value of voxels, the FWM method also revealed the particular class toward which the voxel influenced the classification decision. As shown in Figure 1B, regions that discriminate toward resting state (blue) and toward tapping state (yellow/red) are distinguishable. Effectively, FWM revealed a finer delineation of smaller cortical structures than SLD. The FWM method identifies additional regions in the parietal and frontal cortex as discriminative, while in contrast these regions remain undetected with the SLD method. Furthermore, the FWM method specifically identifies the cortical sheet, while the SLD method labels spatially more extended regions reaching deeper than the cortex, and thus including some white matter.

**Figure 2:**
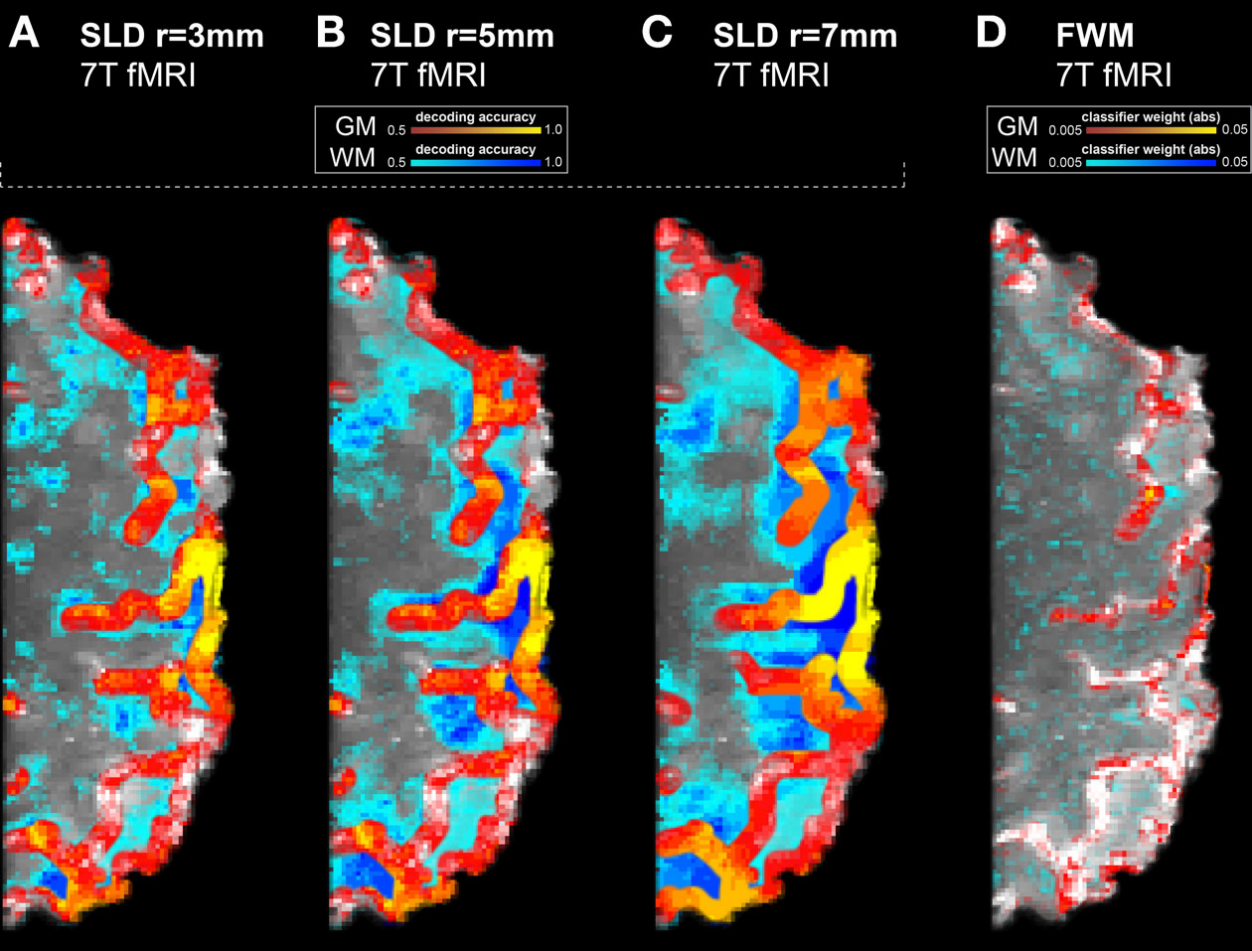
**Results of the high resolution 7T finger tapping data set without multiple comparisons correction, using three searchlight radii and the feature weight mapping method**. White matter voxels are displayed in false colors (by shifting the color hue by 180°). Hence the blue tones indicate false positivity. Dark blue tones indicate high decoding accuracies or high feature weights. **(A)** SLD with a radius of 3 mm. Already at this radius, substantial false positivity is visible on the surface of the cortex on the right side. On the left side, out-of-plane false positivity is visible, as searchlights centered in the selected slice pick up information from the slices below or above. **(B)** SLD with a radius of 5 mm. The levels of false positivity have increased throughout the entire volume. **(C)** With a radius of 7 mm, the SLD method results in substantially inaccurate depictions of true information content. **(D)** Feature weight mapping, to enhance the clarity of the representation only the absolute value of the weights is considered here. The highest (absolute) weights are found within gray matter, while the weights found in white matter are on a low level.

The spatial precision of the SLD method critically depends on the chosen searchlight radius. Larger searchlight radii return accuracy maps where a substantially larger volume is labeled as informative. We depicted three searchlight radii (3, 5, and 7 millimeter) in Figure 2 and contrasted the results with our proposed FWM method. As the two largest searchlight radii failed to reach significance when including multiple comparisons correction, we only show uncorrected accuracy and weight maps. Furthermore, for illustrating the degree of voxels labeled informative within white matter, we applied a gray matter mask: the accuracy or weight of voxels within white matter is displayed in false colors (by shifting the color hue by 180°). We found that for the larger searchlight radii in Figures 2B,C, a substantial number of white matter voxels is indicated with the highest accuracies. In contrast, most highly weighted voxels of the FWM method are found within gray matter.

### Simulated data

Using simulations, we aimed to target how SLD and FWM methods specifically depend on the underlying spatial distribution of information. In total, we created three different levels of coarseness by structuring information in a specific geometry. The searchlight radius was set to three voxels.

**Figure 3:**
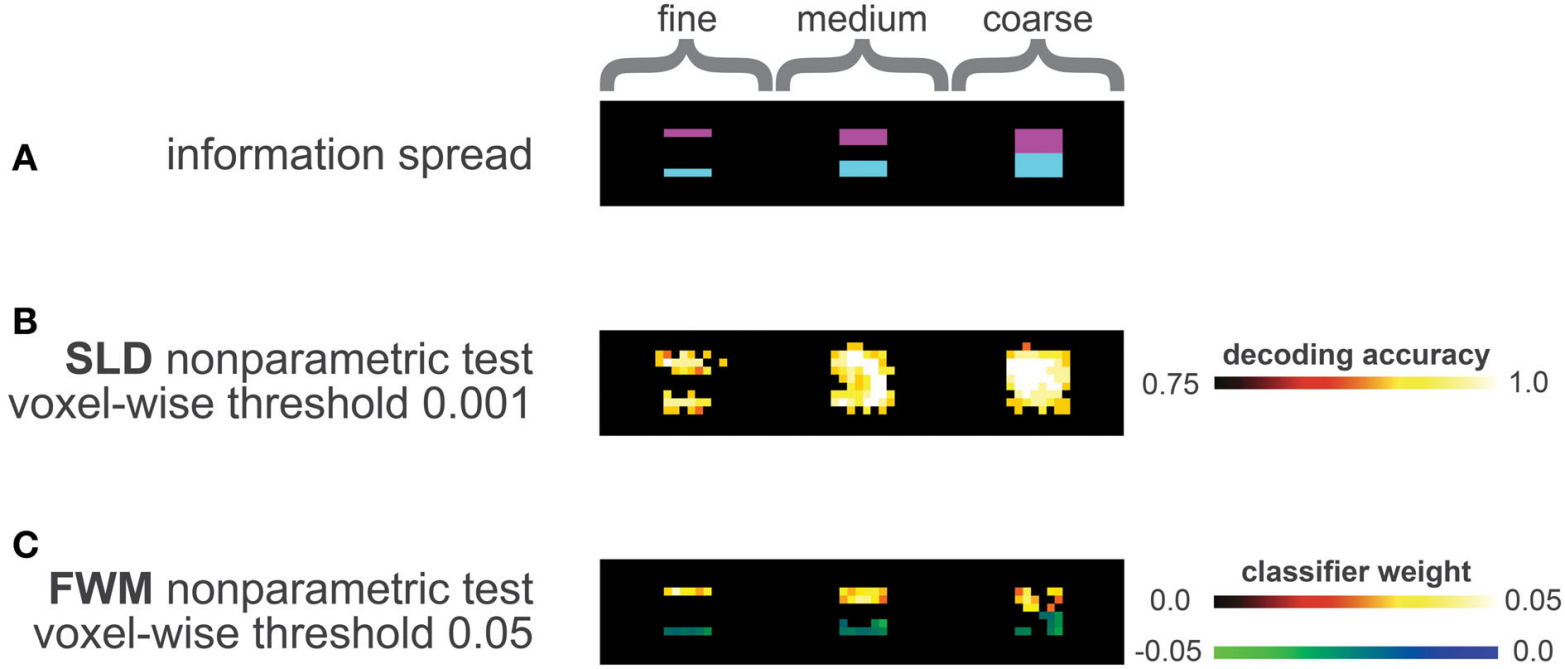
**Analysis of the data simulation (A)** Distribution of information, the three violet half-cubes contained class information for condition A, the three blue half-cubes contained class information for class B. In total, three distinct levels of geometry of information distribution were simulated, the leftmost half-cubes represented a fine information spread, the middle ones an intermediate level and the rightmost half-cubes a coarse information spread. **(B)** Results of SLD method corrected with the non-parametric framework (including multiple comparison correction), using a voxel-wise threshold of *p*_vox_ = 0.001 (one-sided). **(C)** Results of FWM method corrected with the non-parametric framework (including multiple comparison correction) using a voxel-wise threshold of *p*_vox_ = 0.05 (two-sided). The blue-green colors represent influence toward class B, the red colors for influence toward class A.

The information distribution is depicted in Figure 3A, the violet areas represent informative regions of condition A, while the blue areas represent informative regions of condition B.

#### Qualitative comparison of FWM and SLD

Figure 3 depicts the results of applying SLD and FWM on the prepared simulation data.

The SLD method labels most informative regions as significant, while also labeling a considerable number of voxels outside the informative regions significant (Figure 3B).

The tendency for false-positive labeling was especially prominent in the fine and medium distributions, depicted in the left and middle pictures of Figure 3B. Here, the SLD method appeared to overestimate the local information content.

In contrast, the FWM method delineated the informative regions with a high precision (see Figure 3C), and did not label voxels outside of the informative regions as discriminative. The number of true positives, however, was smaller compared to the SLD method, as not all informative voxels were declared discriminative here.

#### Precision and sensitivity

We assessed the statistical performance of the SLD and FWM method by calculating precision and sensitivity curves for each of the three coarseness levels separately. The analysis was carried out with and without application of the multiple comparisons correction.

**Figure 4:**
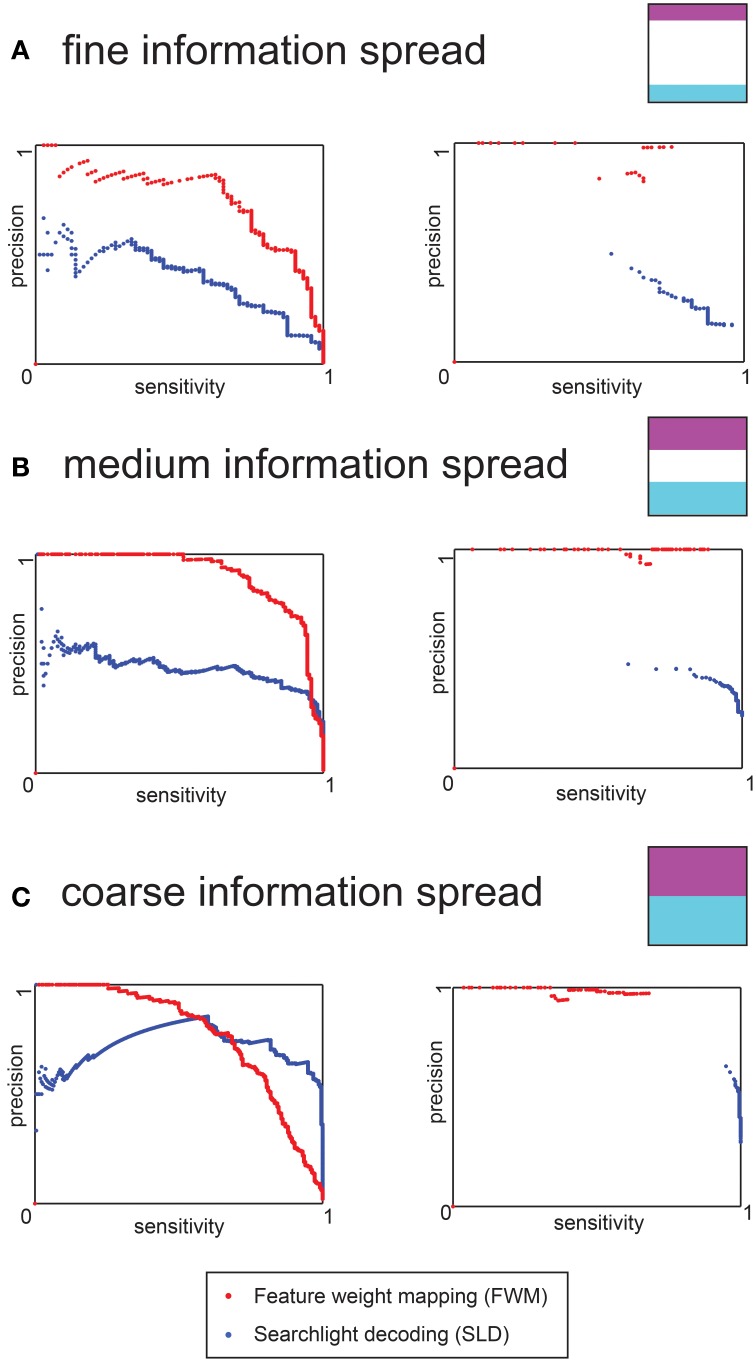
**Precision-sensitivity curves for the three different levels of information distribution of the data simulation**. The red dots represent the FWM method, and the blue dots the SLD method. **(A)** Precision- Sensitivity for a fine information spread. The left chart is based on uncorrected voxel *p*-values (derived from the permutation distribution), the right chart depicts results for the full non-parametric multiple comparison correction **(B)** Precision-Sensitivity for an intermediate distribution of information. The left and right charts are as above. **(C)** Precision-Sensitivity curve for a coarse information spread. The left and right charts are as above.

The three coarseness simulations are depicted separately in Figure 4A (fine information spread), Figure 4B (intermediate) and Figure 4C (coarse information spread). The left charts in Figure 4 depict the analysis without multiple comparison correction while the right charts depict the analysis including multiple comparisons correction.

In the case of the uncorrected charts (left) and for both methods, the sensitivity increased for less stringent (i.e., higher) *p*-values, while the precision declined. For fine and intermediate information spread the FWM method had a higher precision for any given level of sensitivity. Only in the case of coarse information spread and low thresholds (corresponding to high sensitivity) did the SLD method yield a higher precision.

For any given *p*-value threshold, the FWM method and SLD method showed vastly different sensitivity and precision values. While the FWM method performed very well (i.e., high sensitivity and precision) for relatively high *p*-values (e.g., *p*_vox_ = 0.05), the SLD method performed better in the regime of low *p*-values (e.g., *p*_vox_ = 0.001). This difference in optimal choices for *p*-value thresholds was the motivation for the parameters used for the voxel-wise threshold for the fMRI data.

When including the multiple comparison correction, the precision increased substantially for FWM, achieving almost 100% for most cases. Conversely, this precision gain was not found with SLD (right charts of Figure 4. At the same time, FWM never achieved higher than 90% sensitivity, while SLD achieved up to 100% sensitivity, but at considerable loss of precision, in particular for the fine and intermediate information spreads.

## Discussion

Multivariate analysis techniques are commonly regarded as promising candidates for analyzing ultra-high-resolution data acquired with fMRI. In our study we compared two types of multivariate information mapping techniques; the SLD method and our newly proposed FWM method. Both methods (SLD and FWM) aim to determine the local information content in the brain responses elicited by different experimental conditions (hence often termed “information mapping”) and use the same underlying non-parametric framework for statistical analysis, thus both methods are fully comparable.

Using ultra-high-field fMRI data and simulations, we found that our new proposed method (FWM) achieves a considerably higher spatial specificity, (that is to say, a higher accuracy in localization and geometry of information) as compared to SLD. We additionally observed that the results of the searchlight approach were systematically inflated and inaccurate. Notably, SLD mapped information to non-surface cortex regions consisting of white matter. FWM, on the other hand, specifically mapped out the cortical surface. As increasing higher resolution fMRI data become the basis for brain mapping studies, this methodological attention to anatomical specificity is a necessity.

In the following we discuss the peculiarities and differences of both information mapping methods in detail.

### Searchlight decoding

Given that activity-based information in the BOLD fMRI signal is known to be distributed in quite specific types of location in the brain (i.e., within the cortex, small pial veins and subcortical gray matter locations), it should not be surprising that searchlight information maps may be spatially exaggerated and distorted.

**Figure 5:**
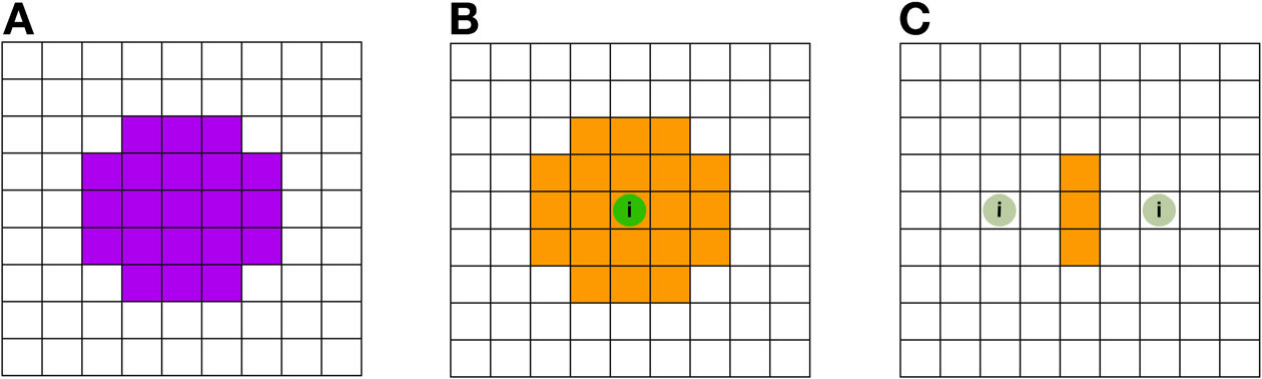
**Schematic illustration of the searchlight induced inflations and spatial inaccuracies. (A)** Searchlight shape (down-projection to 2D) with a 5-voxel diameter. The violet shaded voxels are located within the searchlight. **(B)** No voxels carry class information, except the center voxel featuring the green sphere labeled with the letter “i”: this voxel is the sole voxel carrying class information. As a result of the SLD procedure using searchlight decoding **(A)**, many voxels are being labeled as informative (these voxels are depicted in orange). The inflating effect has previously been termed as “needle in the haystack effect” (Viswanathan et al., 2012). **(C)** Here, no voxels except the two voxels with the green sphere labeled with “i” carry class information. The information carried by one voxel, however, is sufficiently small so that a searchlight has to include both informative voxels in order to be labeled significant. Hence only the voxels in the middle, where the searchlight contains both informative voxels, are labeled informative, resulting in inaccurate and distorted information maps.

Let us consider for example an image containing no signal except one center voxel containing a large amount of class information. The resulting searchlight information map (depicted in Figures 5A,B) will label every searchlight location which contains this center voxel as “informative,” effectively grossly inflating the actual informative regions—thus giving many false positive attributions. In a recent study, this effect has been termed a “needle in the haystack effect” (Viswanathan et al., 2012). Another effect can also be considered in an image containing only two low-informative voxels. The direct area around each voxel will be mapped uninformative by the searchlight approach, and will thus appear as false negative, while those searchlights that contain *both* informative voxels will be labeled informative (depicted in Figure 5C). Thus a distorted picture of the geometry of information is provided by SLD. Appropriately, this effect had been given the name “haystack in the needle” (Viswanathan et al., 2012).

It is easy to see that the latter effect depends on the searchlight diameter, as the number of informative voxels monotonically depends on the diameter of the searchlight. However, the effect also depends on the distribution of information and overall geometry. Lastly, the (multivariate) signal to noise ratio presumably also plays an important role.

Because one main benefit of ultra-high-field fMRI is that it allows the study of activations at fine spatial scales (and so help to establish structure-function relationships), it thus appears questionable whether the searchlight is the optimal method of choice (Kriegeskorte and Bandettini, 2007).

The results of our case study and simulations fully supported the above considerations with regard to exaggeration of spatial extent and other spatial inaccuracies. The searchlight method indeed yielded inflated estimates of information distribution in both the simulated and ultra-high-field fMRI data set. SLD labeled a high fraction of voxels as informative outside gray matter areas, obscuring the actual distribution of information in the cortex (Figure 2A). This issue becomes especially predominant for larger searchlight radii, such as five and seven millimeters (Figures 2B,C). It should be noted that searchlights of such dimensions are common practice, or even exceeding seven millimeters (Soon et al., 2008; Stelzer et al., 2013). The simulations reflected the same spatial inaccuracies of the searchlight method as found previously in our fMRI data. In here, we were able to modify the underlying spatial geometry of the true information distribution. We found that the spatial inaccuracies of the resulting information maps were especially visible in areas of information distributed on small spatial scales (Figure 3B).

The effect was especially pronounced in cases where adjacent informative regions were separated by a small uninformative layer (middle column of Figure 3B). Here, the searchlight method labeled the uninformative border region as highly informative.

The issue of exaggeration of spatial extent (“inflation”) may be mitigated by limiting the searchlight only to gray matter voxels or even directly applying it on the cortical surface (Chen et al., 2011). However, for the surface-based methods, inflation is only reduced in one of three spatial dimensions; while the spatial accuracy in the direction normal to the cortical surface is improved, the two in-plane dimensions (along the cortical sheet) remain inflated and distorted.

Another issue that needs to be addressed is the claim that the searchlight method is only sensitive to local patterns, because it analyzes only a small neighborhood of voxels at a time. The searchlight method is often considered advantageous when it comes to fine-grained *local* representations, where the information is contained in a small region including only few voxels. While this argument may hold for a single searchlight location in isolation, the argument does not necessarily carry over to a searchlight *map* consisting of many searchlight locations. Due to the inflationary nature of the searchlight's information maps, small informative regions will be contained in *many* searchlight locations. Ultimately, the representation of the information content is hence inflated to a degree where small representations either fall under the statistical threshold (when including a whole-brain correction) or are grossly overestimated in their spatial extent (see Figures 1, 4). Furthermore, the intrinsic “smoothing” effect of the searchlight method may be severely exacerbated by the inclusion of spatial smoothing as a post-processing step (e.g., group-level comparisons).

From a conceptual point of view it can be argued that the searchlight's exclusive sensitivity to local patterns may provoke an unrealistic impression of brain function, given that the brain is a large and massively interconnected network. It is most likely that the fingerprint of distinct brain states does not solely exist at small spatial scales. Instead, the brain processes information on larger spatial dimensions across wide-spread networks. For instance, remote brain areas have been observed to jointly exhibit patterns of activation governed by long-range neural communication (e.g., Laughlin, 2003). Evidently, such large-scale interactions cannot be captured by the searchlight method. Although sometimes a strictly local investigation at small spatial scales is desired, for example in (Diedrichsen et al., 2013), it is not clear that the searchlight method is even suitable for such studies, given its potential for false positive and false negative attributions of informative voxels.

### Feature weight mapping

The FWM method is a global multivariate information mapping technique based on dimensionality reduction, which comprises a support vector machine classifier and subsequent non-parametric statistical analysis. Ultimately this allows computation of feature importance and includes multiple comparison correction. The FWM method is tailored for the analysis of extremely high-dimensional data such as that produced by high-field fMRI while yielding spatially precise information maps.

We found that FWM consistently yielded fine-structured information maps. In 7T fMRI data FWM revealed informative regions precisely within the thickness of the cortex. Compared to SLD, FWM labeled uninformative regions (e.g., within white matter) much less often as significant.

In simulations, it delineated the informative regions precisely. Precision and sensitivity curves were generally better for FWM than for SLD, when the spatial distribution of information was within the fine and intermediate information spread range. For any given value of sensitivity (detected informative volume), FWM was more precise than SLD (less false positivity). In the simulations, FWM never reached the highest sensitivity levels (>90%), which were accompanied by an extreme loss in spatial precision in the SLD method.

Another advantage of FWM over SLD is the sign of the mapping, which reveals the particular class to which the voxel influences the classifier. For instance, if a voxel activates consistently when in class A but does not activate when in class B, the resulting weight component would be positive. On the other hand, if the voxel activates consistently when in class A but does not activate when in class B, the weight component would be determined negative. Hence, the individual weight mapping reveals how the corresponding voxel influences the classification decision depending on the level of activation found in the feature. In an area with positive weights, a high activity level would influence the classifier to decide on class A, while a low level of activity would indicate class B. For negative weights, an analogous argument can be made: Here a high level of activity would influence the classifier to decide on class B and a low level of activity would indicate class A. In contrast, the SLD method is unable to deliver such information about the direction of influence for any given features, as it only determines whether class information is present or not.

In contrast to SLD, FWM is a truly global multivariate approach, that is, it considers all voxels simultaneously. Since the classification has to be computed only once on a relatively small data set (for each permutation), the computational resources necessary for the non-parametric statistical framework are drastically lower compared to those needed for SLD. Depending on the size of the data set in terms of voxels (resolution) and experimental trials, we found that the computation of the permutations in the FWM method was between 5,000 and 30,000 times faster than the SLD method.

Because the potential for Type I and Type II errors is vastly reduced, the interpretability of FWM-generated information maps is much improved as compared with SLD. With FWM, the influence attributed to a given voxel is solely its influence on the classification of that particular voxel.

Contrastingly, in the SLD method the accuracy at a voxel characterizes the aggregate decodability of a neighborhood of voxels around it. In other words, for the SLD analysis technique, voxels with high accuracies are not necessarily informative themselves; the informative voxels may be located elsewhere in the voxel's neighborhood. This aspect is problematic, as this important distinction is commonly ignored in neuroscience research where searchlight-based analysis is employed (Etzel et al., 2012).

Until now, to our knowledge no method information-mapping method based on multivariate statistic and adapted to ultra-high-field fMRI including a correction for the multiple comparisons problem has been made available to researchers as part of an easy-to-use software package. The proposed FWM method will be made available as part of LIPSIA (Lohmann et al., 2001) for free use.

### Conflict of interest statement

The authors declare that the research was conducted in the absence of any commercial or financial relationships that could be construed as a potential conflict of interest.
